# Real-time Detection of Breast Cancer Cells Using Peptide-functionalized Microcantilever Arrays

**DOI:** 10.1038/srep13967

**Published:** 2015-10-05

**Authors:** Hashem Etayash, Keren Jiang, Sarfuddin Azmi, Thomas Thundat, Kamaljit Kaur

**Affiliations:** 1 Faculty of Pharmacy and Pharmaceutical Sciences, University of Alberta, Edmonton, Alberta, T6G 2E1, Canada; 2 Department of Chemical and Materials Engineering, University of Alberta, Edmonton, Alberta, T6G 2V4, Canada; 3 Chapman University School of Pharmacy (CUSP), Harry and Diane Rinker Health Science Campus, Chapman University, Irvine, California, 92618-1908, USA

## Abstract

Ligand-directed targeting and capturing of cancer cells is a new approach for detecting circulating tumor cells (CTCs). Ligands such as antibodies have been successfully used for capturing cancer cells and an antibody based system (CellSearch^®^) is currently used clinically to enumerate CTCs. Here we report the use of a peptide moiety in conjunction with a microcantilever array system to selectively detect CTCs resulting from cancer, specifically breast cancer. A sensing microcantilever, functionalized with a breast cancer specific peptide **18-4** (WxEAAYQrFL), showed significant deflection on cancer cell (MCF7 and MDA-MB-231) binding compared to when exposed to noncancerous (MCF10A and HUVEC) cells. The peptide-functionalized microcantilever allowed efficient capture and detection of cancer cells in MCF7 spiked human blood samples emulating CTCs in human blood. A detection limit of 50–100 cancer cells mL^−1^ from blood samples was achieved with a capture yield of 80% from spiked whole blood samples. The results emphasize the potential of peptide **18-4** as a novel peptide for capturing and detecting cancer cells in conjunction with nanomechanical cantilever platform. The reported peptide-based cantilever platform represents a new analytical approach that can lead to an alternative to the various detection platforms and can be leveraged to further study CTCs.

*In-vivo* examinations of breast cancer is mainly implemented through techniques like mammography (an x-ray of the breast), ultrasound exams, magnetic resonance imaging (MRI) and/or [18F]fluorodeoxyglucose positron emission tomography, which are typically followed by *ex vivo* biopsy and further checkups^1^. A simple blood test to detect circulating tumor cells (CTCs) that flow in the bloodstream of cancer patients due to cell shedding from primary tumors could complement other detection methods for disease diagnosis. In recent years, molecular and clinical findings have revealed that cancer cells may invade into the blood circulation at early stages of tumor development, emphasizing the importance of sensitive and specific detection of CTCs in the blood^1^. Developing a sensitive and accurate tool for detection of CTCs would provide valuable information on cancer prognosis, diagnosis, monitoring of tumor sensitivity to anticancer drugs, as well as, in personalization of anticancer therapy^1^,^2^.

Numerous approaches have been developed for reliably identifying and quantifying CTCs in blood samples^3^,^4^,^5^,^6^,^7^,^8^. The presence of CTCs or cancer cells in blood (∼hundreds per mL) is masked by normal blood cells that appear at a billion times higher concentration, making their detection challenging. The classical methods for isolation and enumeration of CTCs are time consuming and cannot be used for easy, routine screening to determine disease recurrence and response to treatments. Evolving technologies in the past few years have allowed identification and quantification of CTCs with applicable specificity and sensitivity. Methods such as the immunohistochemistry (IHC)^9^, flow cytometry (FC)^10^ and the polymerase chain reactions (PCR)^11^ are very sensitive and compliant approaches for detections. However, with respect to their applicable use, they continue to suffer from numerous constrains such as the need for the trained cytologist to handle the sample assessments, time-consumption associated with the handling and pre-treatment procedures, as well as the cross-reactivity of the antibodies and nucleotides used during the detections^6^,^12^. Other alternative label-free biosensing technologies to the classical approaches of CTCs detection are under development, such as nanowire sensor^13^, the graphene oxide nano-sheets^14^, the electro-impedance cytometry^15^ and microcantilevers^16^,^17^,^18^. One platform based on the immunomagnetic beads conjugated with an antibody to EpCAM (CellSearch^®^, Veridex^TM^, Warren, PA), is now clinically used for enumeration of CTCs from human blood samples^19^. Majority of these advanced detection platforms rely on antibody and/or oligonucleotide probes for recognition, identification, and quantification of the target cells.

In this study, we report the development of a peptide-based microcantilever array sensor for efficient capture of intact representative cancer cells at low concentrations without pre-requisite labeling or sample processing (Fig. 1). The microcantilever array was functionalized separately with two cancer targeting peptides, namely, a decapeptide **18-4** (WxEAAYQrFL) with an additional C-terminal cysteine or a cyclic RGD peptide (cRGDfC)^20^ using the thiol group of cysteine residue. Peptide **18-4** is a proteolytically stable engineered breast cancer targeting peptide derived from a 12-mer peptide p160 that was identified using *in vivo* phage display for cancer targeting^21^,^22^,^23^. Peptide **18-4** exhibits high affinity for breast cancer cell lines (MCF7, MDA-MB-231, and MDA-MB-435), most likely through a receptor-mediated mechanism, with almost no binding to the noncancerous cells (MCF10A and HUVECs). RGD is a well-studied tumor homing peptide that interacts with specific integrin receptors (αvβ3) overexpressed on several tumor epithelial cells^24^,^25^. However RGD also targets non-tumorigenic tissues as it is recognized by several integrins (8 out of 24 heterodimers) and is therefore deemed less specific. To explore whether cancer cells can be selectively captured with these peptides, breast cancer cells (MCF7 or MDA-MB-231) alone or in combination with non-cancerous MCF10A (derived from the same breast tissue as MCF7) were spiked into a buffer or blood solution to obtain mimics of CTCs in human blood. The cancer cells were detected by recording the nanomechanical bending of the cantilevers in real-time based on the surface stress induced by adhesion of the cancer cells to the immobilized peptides.

## Results and Discussion

### Functionalization of Microcantilevers

**Figure 1:**
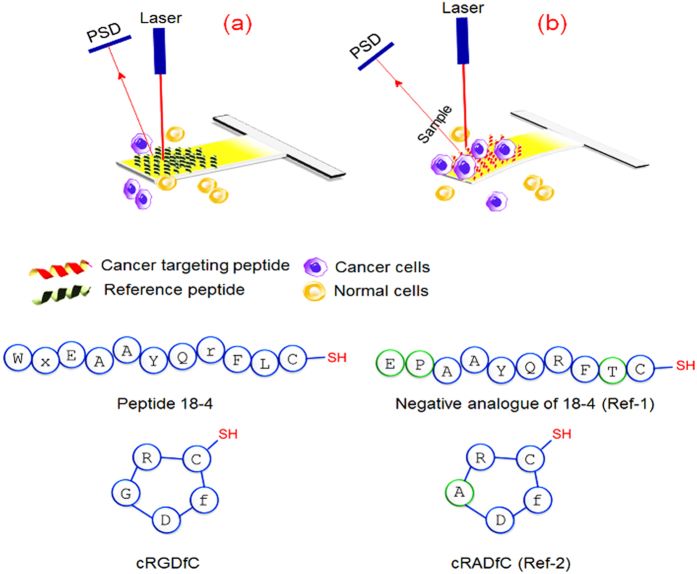
Schematic showing principle of microcantilever sensor operation. (**a**) Microcantilever coated with non-specific reference peptide (Ref-1or Ref-2) shows no response to the presence of normal or cancer cells (no deflection). (**b**) Microcantilever functionalized with cancer targeting peptide (**18-4** or cRGDfC) demonstrates a strong response (deflection) to cancerous cells due to peptide-cancer cell interactions. PSD, Position Sensitive Detector.

Microcantilevers in an array were functionalized with self-assembled monolayers (SAMs) of cancer cell binding peptides, which act as specific ligands for cancer cells. As illustrated in Fig. 1, the detection principle is based on static mode of cantilever operation, which means that cantilever beam bends as a result of changes in the surface stress generated by analyte-ligand interactions^26^. Specifically in this study, selective adsorption of the cancer cells to the immobilized peptide on the surface of the cantilevers results in a decrease in the surface free energy which in turn leads to generate a differential surface stress between the functionalized and non-functionalized sides of the lever. This differential surface stress causes cantilever to deflect or bend by a certain extent that can be expressed according to Stoney’s formula^27^. An in-house built microcantilever array sensor was used for the cantilever experiments (Supplementary Information Figure S1).

One of the essential parameters that determine the efficiency of the cell capture on the microcantilever system is the flow velocity of the sample throughout the system^28^. Therefore, in order to optimize the flow rate, a number of experiments were conducted to determine the sensor capture efficiency at different flow velocities. We spiked cancer cell lines (MCF7) into phosphate buffered saline (PBS) at ∼100 cells mL^−1^ and dispensed on a peptide (**18-4**) coated microcantilever array as a function of flow rates ranging from 1 to 5 mL h^−1^. The capture yield was calculated for each flow rate and results were charted as shown in Figure S2. We found that the estimated capture efficiency increased by decreasing the flow velocity of the samples, indicating an inverse proportion of the capture yield to the sample flow velocity. The capture yield was significantly enhanced at 1 mL h^−1^ flow rate (81%) compared to that at faster flow rate of 5 mL h^−1^ (54%). Based on these results that suggest enhanced binding of the cancer cells to the immobilized peptide with increased incubation time, the subsequent studies were performed using a flow rate of 1–2 mL h^−1^.

### Cancer Cell Binding to the Peptide-functionalized Microcantilevers

**Figure 2:**
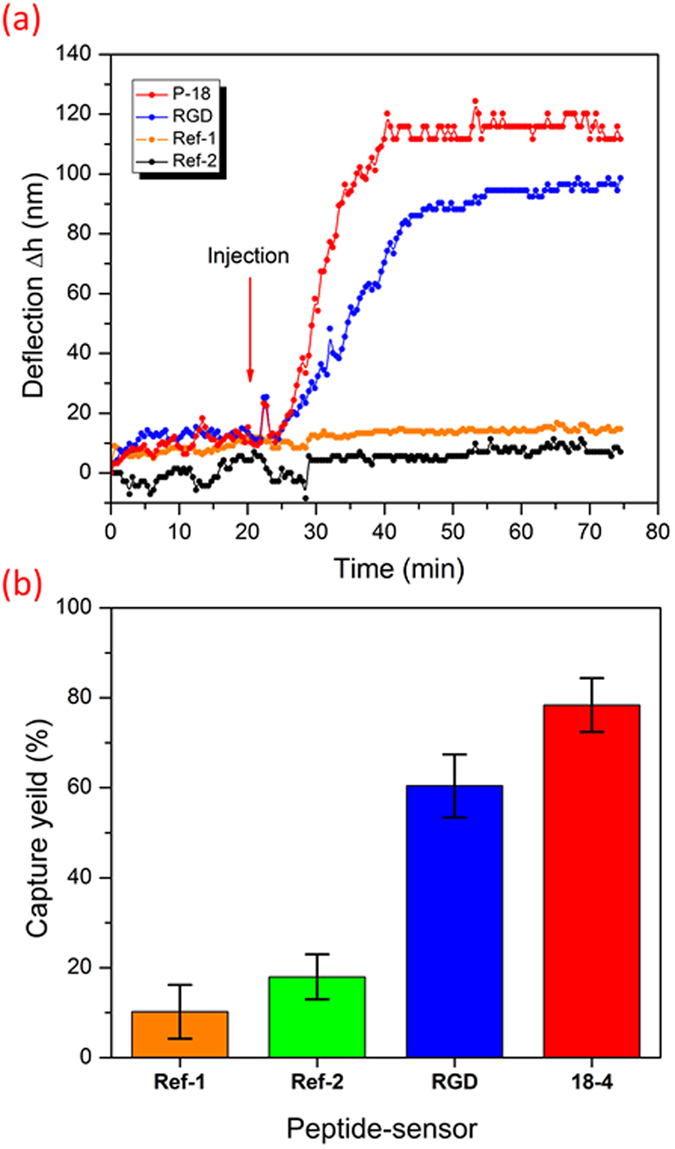
Label-free real time detection of cancer cells using peptide-functionalized cantilever array. (**a**) Real time detection of MCF7 human breast cancer cells mimicking the circulating tumor cells at 25 ± 5 cells mL^–1^ using peptide-functionalized cantilever array. Cantilevers were functionalized with four different peptides, two cancer-targeting peptides (**18-4** and RGD) and two non-specific reference peptides (ref. 1 and ref. 2). (**b**) Capture yield of cancer cells corresponding to each peptide sensor. Each cantilever differential deflection represents an average calculation of eight replicates and error bars indicate standard deviations, *P < 0.05.

First, we aimed to assess and compare the binding efficiency of the designed peptide-based microcantilever sensor (peptide **18-4** sensor) to other peptide-sensors including cRGDfC sensor against the human adenocarcinoma breast cell line MCF7, which is a good mimic for circulating breast tumor cells in human blood. The thiolated peptides (Table S1) were chemically synthesized and independently immobilized on cantilever beams in arrays using the tip-dipping method as described in the material and methods section. Cancer cells were spiked into PBS (25 cells mL^−1^, pH ∼7.4) and were allowed to flow through the microcantilever array. Reference cantilevers functionalized with control peptides were treated with the same concentration of cancer cells and subjected to nanomechanical readings for comparisons. Results of the analysis revealed significant beam deflection for peptide **18-4** functionalized sensor with approximate deflection of 120 ± 7 nm achieved after sample introduction (Fig. 2a). The deflection, however, showed to be slightly less in case of cRGDfC functionalized array with a deflection distance of 102 ± 3 nm. Compared to the peptide **18-4** and RGD sensors, the reference cantilevers (ref. 1 and ref. 2) exhibited insignificant bending when subjected to the cancer cells, indicating weak binding properties of the control peptides.

Further insight on the peptide binding efficiency to cancer cells was gained by estimating the capture yield of cancer cells of each peptide sensor. Figure 2b displays the capture yield (%) of the peptide cantilever sensors in contrast to the reference cantilevers. The calculated capture efficiency was found to be around 80 ± 4% in the case of peptide **18-4** sensor and almost 60 ± 6% for cRGDfC sensor. In contrast to the sensing peptides, the reference cantilevers showed only 14–19% for both ref. 1 and ref. 2. The cantilever results demonstrated that peptide 18–4 sensor has better binding affinity to cancer cell lines than the cRGDfC sensor. These results match well with the previous studies of peptide array whole cell binding assay for screening of cancer targeting peptides using fluorescence microscopy^21^,^22^.

### Specificity of Peptide-functionalized Microcantilevers

**Figure 3:**
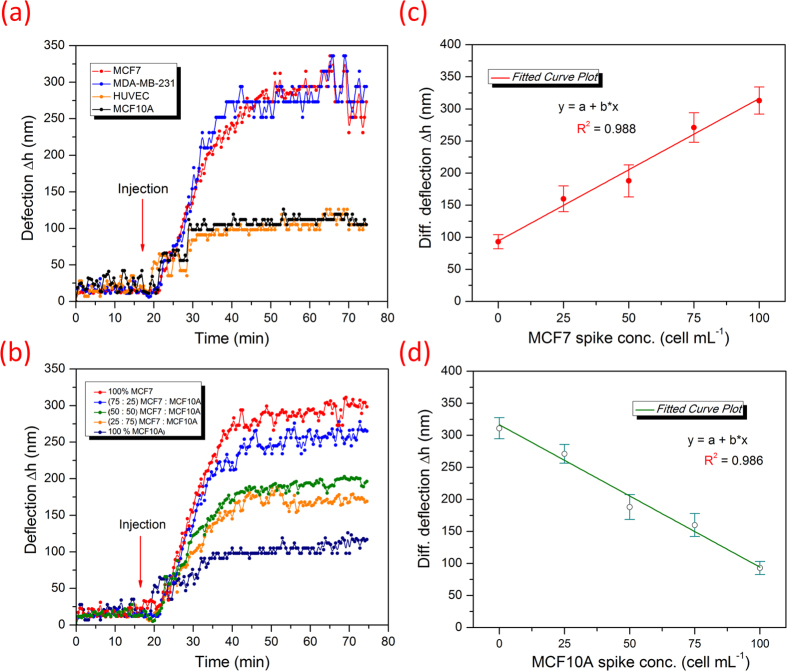
Specific binding of peptide-cantilevers to breast cancer cells. (**a**) Label-free real time recognition of cancer cell lines (MCF7 or MDA-MB-231, 100 ± 10 cells mL^−1^) from non-cancerous cell lines (MCF10A or HUVEC, 100 ± 10 cells mL^−1^) with microcantilever array functionalized with **18-4** cancer targeting peptide. (**b**) Cantilever deflection in response to a function of different concentration ratios of cancerous to non-cancerous cells (MCF7 to MCF10A) in PBS solution as indicated. (**c**) and (**d**) demonstrate the concentration dependence of cantilever response to the number of cancerous or non-cancerous cells, respectively, present in the co-culture sample (100 cells mL^−1^). The representative graphs show an increase in cantilever deflection with an increase in the number of MCF7 cells (**c**), and a decrease in cantilever deflection with an increase in the number of MCF10A (**d**) in the media. Each cantilever differential deflection represents an average calculation of eight replicates and error bars indicate standard deviations.

In order to determine the specificity of the designed sensor, we applied the peptide **18-4** functionalized cantilever array to distinguish between cancerous and non-cancerous cell lines in real-time (Fig. 3). Here the binding affinity of the peptide sensor was explored against two types of breast cancer cell lines, namely, MCF7 and MDA-MB-231 and two non-cancerous cell lines, MCF10A and HUVEC. MCF10A are non-cancerous cells derived from the same human mammary tissue as MCF7, whereas HUVEC are endothelial cells isolated from normal human umbilical vein. When cells were injected at a concentration of 100 ± 10 cells mL^−1^ separately to each peptide cantilever, the cantilever showed significant deflection for cancerous cell binding (280 ± 25 nm) compared to non-cancerous cell binding (90 ± 15 nm, Fig. 3a). The variation in cantilever deflection upon binding cancerous or noncancerous cells is most likely due to the differential expression levels of specific peptide-binding receptors present in cancerous and noncancerous cells. We and others have shown that peptide **18-4** and the original lead peptide p160 enter cells by a receptor-mediated endocytosis^21^,^23^. The receptor is not known yet, however, it is clear that the receptor is overexpressed in breast cancer cells compared to normal cells. The results confirm our conjecture that peptide **18-4** binds breast cancer cells with high specificity. Previously we showed that a similar peptide, peptide **18** (WXEAAYQRFL), binds MDA-MB-435 breast cancer cells with an apparent *K*_d_ of 41.9 *μ*M^22^.

This finding highlights that such tumor binding peptides are not only useful for tumor imaging or targeted drug delivery, but can also be useful as recognition elements to develop peptide-based biosensor platforms for cancer cell detection in real-time. In recent years, several studies have explored the feasibility of using short-ligand peptides as molecular recognition elements in biosensing techniques and have validated the ability of natural and synthetic peptides to serve as robust biorecognition probes in biosensors^29^,^30^,^31^,^32^. We have recently shown that an antimicrobial peptide from class IIa bacteriocins can be used for the detection of Gram-positive *Listeria monocytogenes* at 1 bacterium *μ*L^−1^ using impedance spectroscopy^33^. Furthermore, Mannoor *et al.* showed bioselective recognition of pathogenic bacteria at a single-cell level using peptide assembled onto a wireless graphene nanosensor^34^. Here we have employed a cancer-targeting peptide, engineered from a phage display library and synthetic peptide array library for breast cancer cell binding, as a sensing molecule to detect cancer cells in a cantilever array for the first time.

The differential deflection of the peptide microcantilever sensor to cancer cells was also explored by injecting samples with different ratios of cancer cells (MCF7) to noncancerous (MCF10A) cells. Figure 3b demonstrates cantilevers deflection after injection of cancer cells only (100 cells mL^−1^) as well as after dilution with MCF10A (MCF7:MCF10A; 1:0, 3:1, 2:2, 1:3, 0:1). The cantilevers selectively responded to MCF7 cells and showed amplitude of deflection proportionally scaled with concentration of the MCF7 cells in the sample (Fig. 3c). In a co-culture of cancerous and noncancerous cells, the cantilever was able to detect cancer cells in the presence of ∼75% normal cells (MCF10A). Similarly, as the concentration of normal cells was increased, the deflection signal decreased (Fig. 3d) indicating the ability of peptide probes to discriminate between cell types. The results suggest that the presence of normal cells (MCF10A used here) does not prevent cancer cells from binding to the immobilized probes; it might however, impede their transportation to the sensor probes at lower concentrations dropping the limit of detection. Non-specific binding, sample delivery and improper cell dispersion (mixing) may also contribute to reduced capture sensitivity^35^.

Peptide **18-4** binds to breast cancer cells most likely via a receptor-mediated mechanism^22^. We attribute the variation in cantilever responses between cancerous and non-cancerous cells to the presence of different receptors or different expression levels of a specific receptor on surface of cancer cells. It is well known that certain receptors or/markers are over expressed on cancer cells and deficient in the normal ones^36^,^37^, and such receptors are being targeted for diagnosis and drug delivery using different types of ligands such as antibodies, aptamers, affibodies and peptides.

The sensitivity of detection is one of the key features for practical application of the sensor in medical and biological applications. To this end, the sensitivity of the peptide based cantilever array was determined by exposing the sensor to various concentrations of cancer cells (MCF7) spiked in PBS (5 ± 3–10^3^ ± 10 cells mL^−1^) (Figure S3). The results showed the ability of sensor to detect as low as 25 ± 5 MCF7 cells per mL in pure buffer solution from the background deflection (baseline). The signal, however, was not distinguishable from the background at lower concentration, suggesting a minimum detection limit of 25 cells per mL. Several studies have shown that biosensor performances are often affected by the analyte transport in the vicinity of the sensing area^28^,^38^ as well as dispensing of the cells in the microfluidic system^35^,^38^. Therefore in flow through systems like microcantilevers, it is possible to achieve a low detection limit by controlling the fluid delivery with a proper mixing regime. In addition, cantilever with a continuous fluidic flow can allow analysis of relatively large sample volumes with a few CTCs, thereby improving the detection limit, as opposed to other detection methods with fixed sample volumes.

### Cancer Cell Binding using Surface Plasmon Resonance

**Figure 4:**
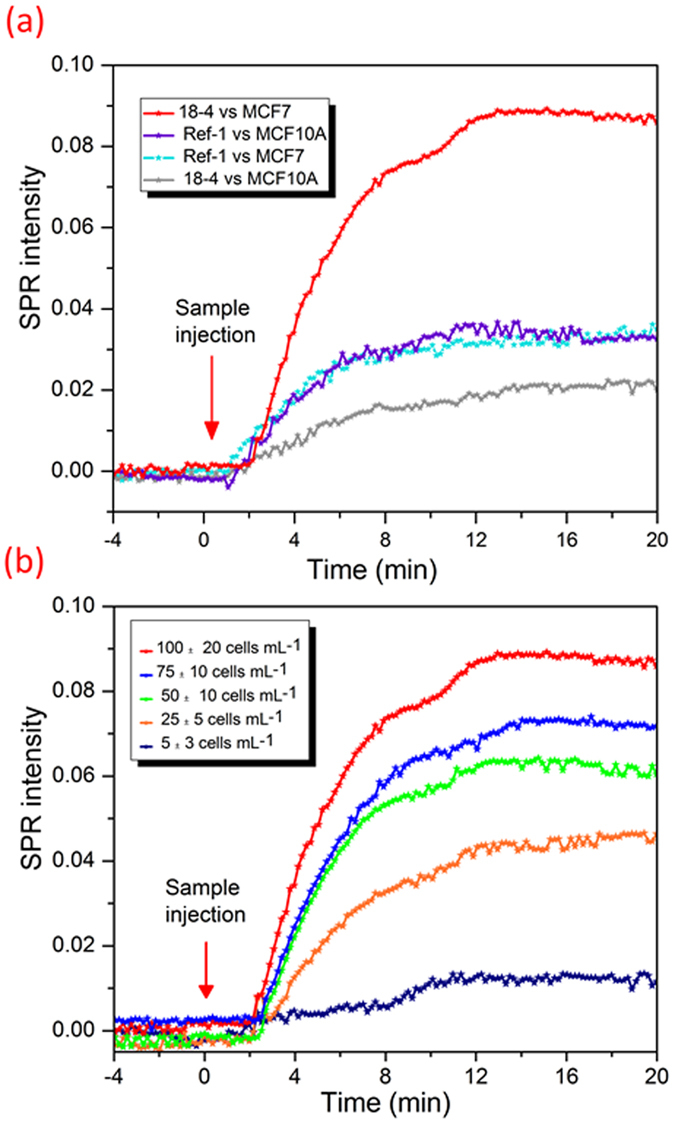
Direct discernment of cancer cells from non-cancerous cells by peptide-based SPR sensor. (**a**) SPR intensity signal resulting from interaction of **18-4** functionalized SPR chip (or functionalized with reference peptide) with MCF7 or MCF10A cells. (**b**) SPR sensitivity spectra for peptide **18-4** against various concentrations of cancer cells (MCF7) at a constant flow rate of 10 *μ*L min^−1^.

In order to validate microcantilever results, surface plasmon resonance (SPR) was utilized to study the specific recognition of cancer cells by surface immobilized cancer targeting peptides. SPR is routinely used as a standard characterization tool for bimolecular interactions and serves as a complementary transduction method to the piezoresistive microcantilever system^39^. SPR is a highly sensitive method, however, piezoresistivity, the change in electrical resistivity under stress or deflection is a simple method that eliminates the complexity inherent to optical instruments such as SPR without the loss of sensitivity. The ligand peptide **18-4** was covalently immobilized on SPR gold slide using the thiol chemistry as described above. The peptide functionalized slide was inserted into the instrument and PBS solution was allowed to flow at a constant flow rate of 10 *μ*L min^−1^. SPR slide functionalized with a reference peptide (ref. 1) was used at the same time on another SPR channel for comparison. The sensor selectivity to the target cells was measured by SPR reading after injecting samples of cancerous (MCF7) and noncancerous cells (MCF10A) simultaneously at a concentration of 100 cells mL^−1^. Figure 4a displays a typical SPR spectrum illustrating responses of the SPR sensor functionalized with peptide **18-4** or ref. 1 to MCF7 or MCF10A cells. A sharp SPR signal was generated for specific interaction between the peptide **18-4** sensor and MCF7 cancer cells compared to the other signals. A low response was observed for peptide **18-4** binding to MCF10A cells followed by similar response signals by the reference sensor to both MCF7 and MCF10A cells. The responses, however, are likely related to non-specific interactions with the sensor surface since no clear differentiation exists between the two cell lines. In agreement with the cantilever results, peptide **18-4** SPR sensor exhibited highest signal to MCF7 cells indicating a specific interaction to the corresponding cells and confirming the applicability of the assay to distinguish between cancerous and noncancerous cells in real-time.

The sensitivity of the peptide functionalized SPR sensor was evaluated by injecting serial concentrations of MCF7 cancer cells (5 ± 3 to 100 ± 10 cells mL^−1^) to the peptide **18-4** sensor at a fixed flow rate of 10 *μ*L min^−1^. Figure 4b shows a representative SPR spectrum where an increase in SPR intensity was observed with an increase in concentration of the injected MCF7 cells. Similar to the microcantilever studies (Figure S3), the number of cells bound to the immobilized ligand is directly proportional to the number of cancer cells in the sample, up to a maximum of ∼500 cells mL^−1^, where the saturation takes place.

### Cancer Cell Detection in Whole blood Samples

**Figure 5:**
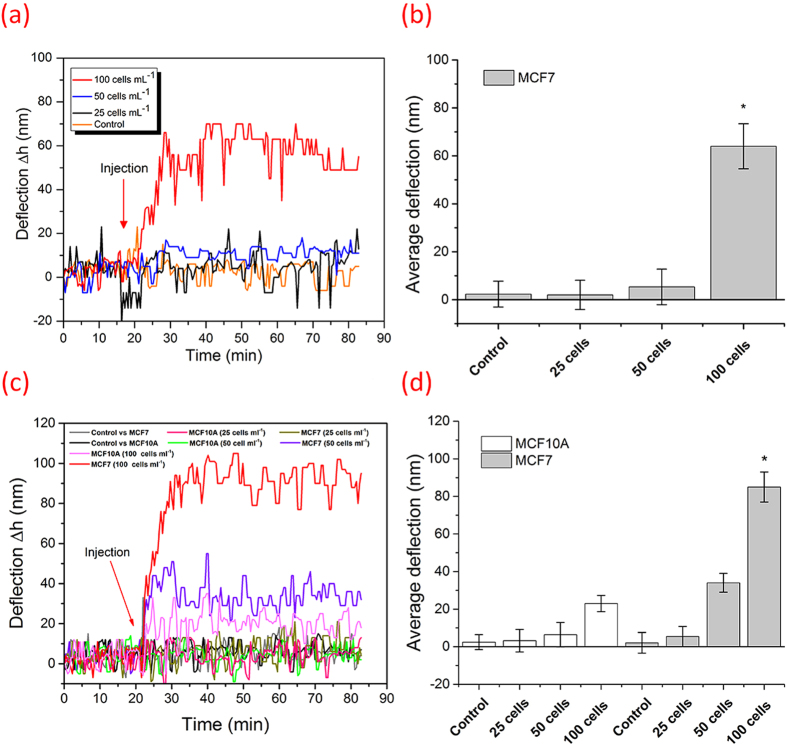
Differential deflection of microcantilever arrays with MCF7 spiked into human blood samples. (**a**) Top figure shows results from injection of whole blood spiked with MCF7 cells, whereas (**c**) lower figure shows data from injection of blood without plasma where blood was first spiked with MCF7 or MCF10A cells followed by plasma removal. The system was first equilibrated by injection of blood samples free from cell lines, followed by injection of samples containing 25, 50 or 100 cells/mL. The control represents the response of a negative analogue of peptide **18-4** to ∼100 cells mL^−1^ spiked samples. Figures (**b**) and (**d**) show the average deflection of peptide **18-4** coated cantilevers in both, whole blood and blood without plasma, respectively, based on three individual studies performed under the same conditions. The error bars indicate corresponding standard deviations.

To mimic the detection of CTCs from patient blood samples, the designed peptide-based microcantilever were exposed to MCF7 cells spiked in human blood samples. First, the blood was made less viscous by diluting it with buffer solution (90%) in order to facilitate the injection and diminish the viscosity effects. In addition, to enhance the sensitivity, the plasma was removed from the blood by centrifugation. Plasma is routinely removed from the blood for CTC enrichment from whole blood^40^,^41^. Blood samples spiked with different concentrations of MCF7 (25, 50 or 100 cells/mL) were allowed to flow over the peptide **18-4**-cantilever (Fig. 5a,b). Likewise for blood without plasma samples, freshly obtained blood samples were first spiked with MCF7 or MCF10A (25, 50 or 100 cells/mL) followed by plasma removal. The resulting sample was allowed to flow over the peptide **18-4**-cantilever for nanomechanical readings (Fig. 5c,d). The cantilever system was initially equilibrated by injecting the blood sample (with or without plasma) free from the cell lines, followed by injection of spiked blood samples. This was done to clearly observe the deflection after introduction of the spiked blood. Instead when the system was equilibrated by injecting PBS, followed by blood and then spiked blood, the deflection due to spiked blood was less apparent (Figure S4). We envisage the patient samples can be run in the clinics by equilibrating the system with normal human blood followed by injection of the patient blood in the cantilever flow through system to obtain a clear read out. Figure 5 shows the differential deflections of microcantilever arrays after injecting the blood (Fig. a,b) or blood without plasma (Fig. c,d). An increase in cantilever deflection was observed with an increase in number of MCF7 cells in the sample. The MCF7 spiked blood samples, whole blood or blood without plasma, showed a substantial deflection compared to the MCF7 free specimens at a concentration of 100 ± 10 cancer cells mL^−1^. Blood without plasma showed higher response (85 ± 8 nm) compared to whole blood sample (62 ± 10 nm). In addition, for the blood without plasma specimen (Fig. 5c) the nanomechanical bending was significant (40 ± 10 nm) even at 50 ± 10 MCF7 cancer cells mL^−1^, suggesting interference from plasma components such as proteins and other interfering biomolecules.

**Figure 6:**
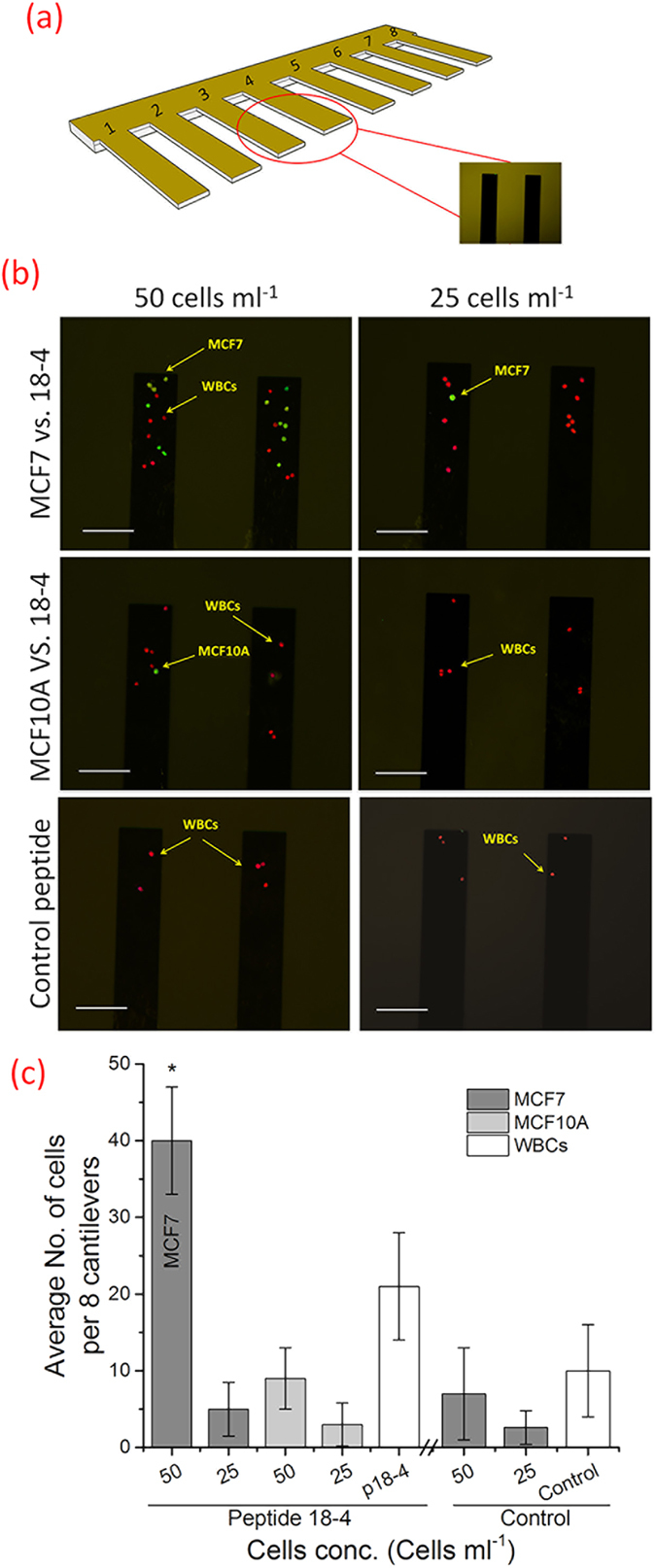
Cancer cell capture on the peptide coated microcantilevers (eight) using fluorescence microscopy. (**a**) Schematic of 8 cantilevers used to capture cancer cells when exposed to 50 or 25 cells/mL sample. (**b**) Fluorescence microscope images of the captured cells (MCF-7 or MCF10A) on the peptide-coated (18-4 or control) microcantilevers (two of the eight cantilevers are shown here). The eight cantilevers were exposed to MCF7 or MCF10A spiked whole blood samples (50 or 25 cells/mL). MCF7 or MCF10A cells and the white blood cells (WBCs) were stained separately, green and red, respectively, before mixing. Scale bar is 100 *μ*m. (**c**) The average number of captured cells per 8 microcantilevers as computed from the set of fluorescence microscopy images. Assay was repeated five times, and mean ± SD is presented. The P values were computed using the unpaired *t*-test to signify the statistical difference between the comparable groups.

Further, we examined the cancer cell capture from whole blood samples using fluorescence microscopy with comprehensive image analysis. MCF7 (or MCF10A) and white blood cells (WBCs) in blood samples were fluorescently labeled green and red, respectively, followed by injection into the cantilever. The eight microcantilevers were exposed to two spike cell concentrations, 50 or 25 cells mL^−1^ of blood at 1 mL hr^−1^ (Fig. 6a). The captured cells were imaged using fluorescence microscopy. Figure 6b shows images of two of the eight cantilevers with captured MCF7 (or MCF10A) and hematological cells (WBCs). While the control peptide cantilevers captured almost no cancer cells, the peptide 18-4 cantilevers captured 5 ± 2 MCF7 cells/cantilever. Overall, the average number of captured MCF7 cells per 8 cantilevers, when seeded at a concentration of ∼50 cells/mL, was found to be 40 ± 7 cells/mL for the peptide **18-4** coated sensor which is significantly higher than the control sensor (7 ± 5 cells/mL). These results are statistically significant as determined using the unpaired student t-test (P = 0.008). In contrast, at seeded concentration of 25 cancer cells/mL, poor significant difference was observed for the peptide **18-4** sensor compared to the control sensor (P = 0.056; *n* = 5). In addition, peptide **18-4** coated sensor captured only 9 ± 3 cells/mL MCF10A cells when exposed to ∼50 MCF10A cells/mL in blood sample. From the optical images of single cells (MCF7) bound to microcantilevers, we can clearly see the results correlate reasonably well with the cantilever deflection measurements (Fig. 2b). An increase in bound cancer cells was observed with an increase in its seeded concentration. At 50 cells/mL, high capture efficiency (80 ± 5%) was achieved using peptide 18-4 functionalized microcantilevers. It is interesting to note that a similar capture yield (80%) was obtained for MCF7 cells present in PBS (Fig. 2b) or blood samples (Fig. 6c), whereas the cantilever deflection was decreased when MCF7 cells were present in whole blood (Fig. 5a) compared to when present in PBS (Fig. 2a). This is likely due to the higher baseline deflection for the spiked whole blood sample, where other biomolecules or cells from the blood bind to the peptide cantilever before cancer cell binding. For instance, we observe that white blood cells contribute to the non-specific binding on the peptide **18-4** cantilevers with 21 ± 6 WBCs bound per 8 cantilevers.

Owing to the high specificity of peptide **18-4** to breast cancer cells, detection of cancer cells was achieved in buffer and blood samples at reasonable concentration levels. Although it has been very challenging to detect cancer cells in pure blood samples, we achieved the detection limit of approximately 50 cell mL^−1^, which compares well with other reported data^13^. Typically, antibodies or nucleotides are used as molecular recognition elements in cancer detection^42^,^43^. The only test that has been approved by the US Food and Drug Administration to measure CTCs in patients is the CellSearch^®^ system (Veridex^TM^, Warren, PA). This system is based on epithelial cell adhesion molecule (EpCAM) recognition by anti-EpCAM antibody^41^. The system is very sensitive, achieves robust capture efficiency, and is used for clinical prediction of CTCs with enumeration count of 5 or more cancer cells per 7.5 mL human blood. It is reported, however, that even with CellSearch a number of cancer cells escape from the detection due to the lack of the EpCAM molecule or due to the multiple steps that are required for the enrichment process^12^. There are several other platforms that use EpCAM antibody and are under development, such as nanowire based platform and platinum microelelctrodes coupled with electrochemical impedance^44^,^45^. Lee and co-workers reported an integrated nanowire based platform where the EpCAM antibody immobilized in the quartz nanowire arrays captures CTCs from blood samples and laser scanning cytometry is used to enumerate the CTCs^44^. Similarly, in another study EpCAM antibody is used to capture cancer cells and the binding event is monitored using highly sensitive electrochemical impedance sensor^45^. In this case, however, the detection sensitivity is dependent on the ionic strength of the sample and the frequency at which the electrical impedance is measured.

More recently, a genetic-based approach is reported where nanoconstructs called “NanoFlares” are used to detect live circulating tumor cells from blood^46^. NanoFlares consist of gold nanoparticles functionalized with single-stranded DNA (antisense recognition motif) that binds to short DNA complement containing a fluorescent reporter, whose fluorescence is quenched when it is present near the gold particle. In the presence of cancer cells the NanoFlares bind to target mRNA, and the fluorescent reporter is away from the gold nanoparticles displaying enhanced fluorescence which is quantified using flow cytometry. Other methods for CTC detection include capturing CTC based on the cell size difference^47^,^48^. CTCs are typically larger than peripheral blood cells and different filtration approaches are being developed to isolate and detect CTCs^47^.

Our study explores an alternative selective biomolecule (peptide) to detect cancer cells in combination with highly sensitive microcantilevers. The technique not only detects cancer cells by peptide capture, it also sorts cells in a single step. Unlike other techniques, peptide-based cantilever arrays are very simple to prepare, can be readily fabricated on silicon wafers and/or other materials using conventional microfabrication techniques, are inexpensive and can be used in an array format to detect simultaneously several cancer phenotypes. The ultra-small size of cantilevers, which resembles a miniature diving panel, allows the sensor to exhibit quick responses to the biological and chemical deviations for real-time, *in-situ* monitoring^49^. Peptide functionalized cantilever arrays can be developed to capture multiple receptors expressed on cancer cells increasing sensor sensitivity. Our future work will focus on obtaining a well-defined peptide array with different binding affinities for cancer cells and the normal haematological cells in blood samples. The approach will encompass exploring strategies such as employing different techniques for peptide immobilization, investigating multi-ligands for targeting and using other sensor platforms in parallel to achieve better detection limits with high selectivity. Peptide **18-4** works well to capture the immortalized cells spiked into human blood. Future work warrants the evaluation of peptide **18-4** binding to patient derived CTCs to validate the peptide-based microcantilever approach. Currently peptides are being used clinically to detect cancer^50^,^51^,^52^. For instance, RGD that binds αvβ3 integrins on cancer cell surface is used in cancer patients as a radiotracer to detect breast cancer lesions by positron emission tomography (PET)^50^.

In conclusion, functionalization of microcantilevers with breast cancer-targeting peptide **18-4** has enabled label-free sensing platform for real-time detection of cancer cells in human blood samples. The peptide **18-4** functionalized cantilever sensor can detect cancer cells in whole blood which contains significantly large number of hematological cells. The achieved detection limits with the cantilever sensor are 25, 50, and 100 cancer cells/mL in buffer, blood without plasma and blood, respectively. The higher sensitivity toward the blood without plasma sample suggests that the microcantilever sensing can be further improved by removing the non-cellular components from the blood. Further a capture yield of 80% from spiked whole blood samples was achieved with the peptide **18-4** functionalized cantilevers, which is comparable to the antibodies based systems^14^. These results suggest that the peptide-based microcantilever sensor can be developed into a diagnostic platform for detection of circulating tumor cells as well as to monitor the therapeutic outcomes in cancer patients.

## Methods

### Peptide Design and Synthesis

Two cancer-targeting peptides, peptide **18-4** (WxEAAYQrFLC) and cRGD (cyclicRGDfC) and the corresponding negative control peptides, ref. 1 (XEPAYQRFTC) and ref. 2 (cyclicRADfC) were used in this study. In each peptide an additional cysteine residue has been added at the terminus in order to enable adequate anchoring to the cantilever gold interfaces and the SPR gold chips through the well-known gold-thiol chemistry immobilization method^20^,^53^ Peptides were synthesized chemically using standard N-Fmoc solid phase peptide synthesis as described previously^21^. Briefly, the first amino acid was coupled to a 2-chlorotrityl resin (NovaBiochem, San Diego, CA) at 5-fold excess using the N,N diisopropyl ethylamine (DIPEA) at room temperature. Further amino acids were added automatically using an automated peptide synthesizer (Tribute, Protein Technology, Inc., USA). The completed peptide was ultimately released from the resin with a mixture of 90% trifluoroacetic acid (TFA), 9% dichloromethane (DCM), and 1% triisopropylsilane (∼10 mL) for 90 min at room temperature. The cleaved peptide combined with TFA was then concentrated, washed with diethyl either, dissolved in water and purified using reversed-phase HPLC (Table S1).

### Microcantilever Sensor Preparation

Microcantilever arrays (Concentris GmbH – Switzerland) of eight gold-coated cantilevers (500 *μ*m long, 100 *μ*m wide and 1 *μ*m thick) were used in the experiments. The apex – top gold surfaces of the cantilevers (20 nm gold thickness) were functionalized with our designed thiolated peptides following the procedure described for gold-thiol chemistry immobilization^20^,^53^. Briefly, cantilevers were cleaned with Piranha solution (30% H_2_O_2_:96% H_2_SO_4_, vol/vol) for 15 minutes, rinsed three times with MilliQ-water (18 MW) followed by ethanol, and dried in air. The arrays were incubated in 2-[methoxy(polyethyleneoxy)propyl]trimethoxysilane (10 mM, Gelest Inc. Frankfurt, Germany) for 20 minutes, rinsed with ethanol and dried in air in order to make the backside of the levers inert and reduce nonspecific binding to the silicon side. Subsequently the microcantilevers were coated with the peptides of interest. In order to make sure that only cantilevers tips were functionalized with the peptide, only the tips were dipped-in the peptide solution (1 mg/mL) and kept for 6 h; the process was also repeated once to ensure an adequate peptide coupling to the cantilever surface. Prior to use, the arrays were rinsed with 70% ethanol and copious amount of PBS solution to remove any physically adsorbed materials.

### Cantilever Setup and Deflection Detection

All cantilever experiments were carried out using an in-house built microcantilever array sensor (Figure S1). Briefly, the cantilever setup consists of a fluidic cell within which the functionalized cantilever array was mounted. The cell is attached to an inlet port connected to a syringe pump for introduction of the sample and an outlet port which is attached to a fluid reservoir. To detect cantilever deflections, a low-power (∼1 mW) laser beam was reflected off the free-end of the cantilever and was focused onto a position sensitive detector (PSD Thorlabs. Inc. New Jersey, USA). Data from nano-mechanical cantilever deflections were recorded in real-time using a multifunctional data-acquisition board driven by LabView-based software. The functionalized cantilever array was initially placed in the fluidic cell and equilibrated in running phosphate buffered saline (PBS) at a constant flow rate of 5 mL h^−1^ until a stable baseline was achieved. It was then exposed to running PBS solution for approximately 50 scans followed by flow of sample solutions containing cancer cells. An optimum flow rate for detection was determined by exposing the peptide **18-4**-functionalized cantilever to a solution of cancer cells at various flow velocities. The results led us to select a flow rate of 1–2 mL h^−1^ for all our subsequent experiments. The experiments were performed for four different peptides as indicated in the text and four different cell lines including the non-cancerous control cells.

### Cell Culture

The human breast cancer cell lines MCF7 and MDA-MB-231 (American Type Culture Collection, Manassas, VA) were cultured in DMEM medium containing 10% fetal bovine serum (FBS), 100 IU mL^−1^ penicillin, and 100 IU mL^−1^ streptomycin. The human mammary epithelial cell line MCF-10A was cultured in minimal essential growth medium (MEGM, Lonza, Cedarlane) supplemented with the same additives as mentioned before. Human umbilical vein endothelial cells (HUVEC), kind gift from the laboratory of Sandra Davidge, University of Alberta, were cultivated using endothelial cell growth medium (EGM, Lonza, Cedarlane) containing 20% FBS, 2 mM L^−1^ glutamine, 100 IU mL^−1^ penicillin, 100 IU mL^−1^ streptomycin, and 2 ng/mL basic fibroblast growth factor (Roche Diagnostics, Mannheim, Germany). All cell lines were cultivated at 37 °C in a 5% CO_2_–95% O_2_ incubator, and growth media were replaced every 48h.

### Cantilever and Cell Capture Assay

For running the cantilever assay and performing the capture efficiency experiments, cells were diluted in serum-free medium, starting at an initial concentration of 10^3 ^mL^−1^ determined by a hemocytometer. Subsequently the cells were centrifuged at 500 rpm, and re-suspended in phosphate buffered saline (PBS, 1 mL). Cells were aliquoted into a low-attachment 96-well plate to obtain a serial dilution of cells ranging from 100–5 cells mL^−1^ in each well (optical microscopy was used for cell counting). Mixed cells samples were obtained by following the same protocol, where aliquots of MCF7 and MCF10A were mixed to give final ratios of 25:75, 50:50, and 75:25 for MCF7:MCF10A with 100 cells mL^−1^ in each well. Before the cell capture assay, MCF7 cells were stained with fluorescent dye (CyQUANT, green – Life Technologies Inc., Burlington, ON, Canada) following the manufacture’s protocol, washed with PBS, centrifuged and re-suspended at 100 cells/mL in PBS solution. The sample with seeded cells was then introduced into the cantilever sensor after calibrating the baseline with PBS solution for ∼20 min. After taking the deflection reading the cantilevers were scanned by a fluorescence microscope (Olympus America, Melville, NY, USA) and sets of images corresponding to the captured cells were taken at different positions. The images were imported and cell numbers were computed using ImageJ software package. The capture efficiency was defined as the ratio of the number of target cells captured to the number of target cells initially seeded.

Human blood samples were collected from healthy donors with informed consent and in accordance with the approved guidelines by the University of Alberta. All experiments were performed following protocols as approved by the ethics committee. All samples were collected in EDTA tubes and were processed within 3 h. Before each injection into the cantilever system, the samples (whole blood or the blood without plasma) were spiked with various concentrations of cells (25, 50, and 100 cell mL^−1^), diluted to 10% concentration in 1×PBS solution, and subjected to nanomechanical reading. Briefly, blood without plasma sample was prepared by first spiking the blood (1 mL) with cell lines (MCF7 or MCF10A), mixed with buffer (1:9, v/v), centrifuged at 800 × *g* for 10 minutes until the blood cells fall to the bottom of the tube^54^ followed by aspiration of the plasma and buffer, and finally re-suspending in 1×PBS solution (1 mL)^40^,^41^. Samples were injected into the cantilever system at a flow rate of 1 ml hr^−1^. The capture yield was determined as discussed above.

The MCF7 or MCF10A and centrifuged white blood cells (from 1 mL blood) were stained green and red, respectively, suspended in PBS solution (1 mL) and injected to the cantilever device. Note that in order to get the red probe (propidium iodide) inside the WBCs, the hematological cells were incubated with the stain for ∼1 h at room temperature. Captured cells were then examined using fluorescence microscopy at 20×magnification and excitation wavelength of 488 nm (CyQUANT) and 543 nm (propidium iodide). The emitted fluorescence was detected through spectral detection channels between 500–530 nm and 555–655 nm for green and red fluorescence, respectively. Capture efficiency was defined as mentioned above.

### SPR Measurements

Surface Plasmon Resonance (SPR) measurements were carried out using a SRP Navi 200 instrument (BioNavis Ltd., Tampere, Finland) that uses the Kretscheman prism configuration having a goniometer with dual flow channels and cohesive peristaltic pump with 100 *μ*L sampler loops. Briefly, the experiments were performed in angular scan mode in order to determine the SPR angular position changes in a real-time. The critical angle of total internal reflection was measured as the reflection index changes due to the surface absorption on the chip. A flow rate of 10 *μ*L min^−1^ was used throughout the experiments with a sensor temperature fixed at 25 °C. A laser with a wavelength of 670 nm was used as a light source to excite the surface plasmon at the dielectric gold interface. A freshly cleaned gold-coated SPR chip (50 nm gold, 5 nm Titanium adhesion layer) was functionalized with peptide **18-4** (or peptide ref. 1) by immersing in peptide/PBS solution (1 mg mL^−1^) for 12 h at room temperature. The measurements started by introducing the peptide chip into the sample holder and running 1×PBS solution at a 7.4 pH to stabilize a baseline. Two samples of PBS (1×) solution, containing cancer cell line MCF-7 (100 cells mL^−1^) or the corresponding normal cell line MCF-10A were injected separately through the flow cell. A continuous scan was performed on a liquid range of 50–77° and the recorded data were processed using the BioNavis software package.

### Statistical Analysis

For all the experiments, signals of identically functionalized cantilevers were averaged and each experiment was performed at least three times. All data are presented as mean ± SD of the calculations throughout the manuscript. The statistical difference was tested either using the unpaired t-test or the one way ANOVA test as specified^55^. In all statistical analysis the significance level (*P* value) was set at 0.05.

## Additional Information

**How to cite this article**: Etayash, H. *et al.* Real-time Detection of Breast Cancer Cells Using Peptide-functionalized Microcantilever Arrays. *Sci. Rep.* **5**, 13967; doi: 10.1038/srep13967 (2015).

## Supplementary Material

Supplementary Information

## References

[b1] Krebs M. G. *et al.* Molecular analysis of circulating tumour cells-biology and biomarkers. Nat Rev Clin Oncol 11, 129–144 (2014). 24445517 10.1038/nrclinonc.2013.253

[b2] Gupta G. P. & Massague J. Cancer metastasis: building a framework. Cell 127, 679–695 (2006). 17110329 10.1016/j.cell.2006.11.001

[b3] Stott S. L. *et al.* Isolation of circulating tumor cells using a microvortex-generating herringbone-chip. Proc Natl Acad Sci USA 107, 18392–18397 (2010). 20930119 10.1073/pnas.1012539107 PMC2972993

[b4] Zemp R. J. Nanomedicine: Detecting rare cancer cells. Nat Nano 4, 798–799 (2009). 10.1038/nnano.2009.367 19966826

[b5] Yu M. *et al.* RNA sequencing of pancreatic circulating tumour cells implicates WNT signalling in metastasis. Nature 487, 510–513 (2012). 22763454 10.1038/nature11217 PMC3408856

[b6] Mostert B., Sleijfer S., Foekens J. A. & Gratama J. W. Circulating tumor cells (CTCs): detection methods and their clinical relevance in breast cancer. Cancer Treat Rev 35, 463–474 (2009). 19410375 10.1016/j.ctrv.2009.03.004

[b7] Wen C. Y. Quick-response magnetic nanospheres for rapid, efficient capture and sensitive detection of circulating tumor cells. ACS Nano 8, 941–949 (2014). 24313365 10.1021/nn405744f

[b8] Alix-Panabieres C. & Pantel K. Technologies for detection of circulating tumor cells: facts and vision. Lab chip 14, 57–62 (2014). 24145967 10.1039/c3lc50644d

[b9] Schoenfeld A. *et al.* The detection of micrometastases in the peripheral blood and bone marrow of patients with breast cancer using immunohistochemistry and reverse transcriptase polymerase chain reaction for keratin 19. Eur J Cancer 33, 854–861 (1997). 9291805 10.1016/s0959-8049(97)00014-2

[b10] He W., Wang H., Hartmann L. C., Cheng J. X. & Low P. S. *In vivo* quantitation of rare circulating tumor cells by multiphoton intravital flow cytometry. Proc Natl Acad Sci USA 104, 11760–11765 (2007). 17601776 10.1073/pnas.0703875104 PMC1913863

[b11] Castells A., Boix L., Bessa X., Gargallo L. & Pique J. M. Detection of colonic cells in peripheral blood of colorectal cancer patients by means of reverse transcriptase and polymerase chain reaction. Br J Cancer 78, 1368–1372 (1998). 9823981 10.1038/bjc.1998.686 PMC2063185

[b12] Gorges T. M. *et al.* Circulating tumour cells escape from EpCAM-based detection due to epithelial-to-mesenchymal transition. BMC Cancer 12, 178 (2012). 22591372 10.1186/1471-2407-12-178 PMC3502112

[b13] Lee S. K. *et al.* Nanowire substrate-based laser scanning cytometry for quantitation of circulating tumor cells. Nano Lett 12, 2697–2704 (2012). 22646476 10.1021/nl2041707 PMC3381426

[b14] Yoon H. J. *et al.* Sensitive capture of circulating tumour cells by functionalized graphene oxide nanosheets. Nat Nanotechnol 8, 735–741 (2013). 24077027 10.1038/nnano.2013.194 PMC4017624

[b15] Choi H. *et al.* A label-free DC impedance-based microcytometer for circulating rare cancer cell counting. Lab chip 13, 970–977 (2013). 23340965 10.1039/c2lc41376k

[b16] Gaitas A., Malhotra R. & Pienta K. A method to measure cellular adhesion utilizing a polymer micro-cantilever. Appl Phys Lett 103, 123702 (2013). 24170959 10.1063/1.4821946 PMC3790771

[b17] Huber F., Lang H. P., Backmann N., Rimoldi D. & Gerber C. Direct detection of a BRAF mutation in total RNA from melanoma cells using cantilever arrays. Nat Nanotechnol 8, 125–129 (2013). 23377457 10.1038/nnano.2012.263

[b18] Shekhawat G. S. & Dravid V. P. Nanomechanical sensors: Bent on detecting cancer. Nat Nanotechnol 8, 77–78 (2013). 23380929 10.1038/nnano.2013.10

[b19] Rasmussen D. J. FDA release letter for CellSearch Epithelial Cell Kit/Cell spotter Analyzer, 2004, http://www.accessdata.fda.gov/cdrh_docs/pdf3/k031588.pdf, date accessed 04/08/2015.

[b20] Zimmermann J. L., Nicolaus T., Neuert G. & Blank K. Thiol-based, site-specific and covalent immobilization of biomolecules for single-molecule experiments. Nat Protoc 5, 975–985 (2010). 20448543 10.1038/nprot.2010.49

[b21] Soudy R., Gill A., Sprules T., Lavasanifar A. & Kaur K. Proteolytically stable cancer targeting peptides with high affinity for breast cancer cells. J Med Chem 54, 7523–7534 (2011). 21967660 10.1021/jm200750x

[b22] Ahmed S., Mathews A. S., Byeon N., Lavasanifar A. & Kaur K. Peptide arrays for screening cancer specific peptides. Anal Chem 82, 7533–7541 (2010). 20799711 10.1021/ac1003085

[b23] Askoxylakis V. *et al.* Characterization and development of a peptide (p160) with affinity for neuroblastoma cells. J Nucl Med 47, 981–988 (2006). 16741308

[b24] Pasqualini R., Koivunen E. & Ruoslahti E. Alpha v integrins as receptors for tumor targeting by circulating ligands. Nat Biotechnol 15, 542–546 (1997). 9181576 10.1038/nbt0697-542

[b25] Desgrosellier J. S. & Cheresh D. A. Integrins in cancer: biological implications and therapeutic opportunities. Nat Rev Cancer 10, 9–22 (2010). 20029421 10.1038/nrc2748 PMC4383089

[b26] Hansen K. M. & Thundat T. Microcantilever biosensors. Methods 37, 57–64 (2005). 16199177 10.1016/j.ymeth.2005.05.011

[b27] Azmi S., Jiang K., Stiles M., Thundat T. & Kaur K. Detection of Listeria monocytogenes with short peptide fragments from class IIa bacteriocins as recognition elements. ACS Comb Sci 17, 156–163 (2015). 25548942 10.1021/co500079k

[b28] Dareing D. W., Tian F. & Thundat T. Effective mass and flow patterns of fluids surrounding microcantilevers. Ultramicroscopy 106, 789–794 (2006). 16650934 10.1016/j.ultramic.2005.11.011

[b29] Mannoor M. S., Zhang S., Link A. J. & McAlpine M. C. Electrical detection of pathogenic bacteria via immobilized antimicrobial peptides. Proc Natl Acad Sci USA 107, 19207–19212 (2010). 20956332 10.1073/pnas.1008768107 PMC2984209

[b30] Wang J. *et al.* Rapid detection of pathogenic bacteria and screening of phage-derived peptides using microcantilevers. Anal Chem 86, 1671–1678 (2014). 24417655 10.1021/ac403437x

[b31] Dhayal B., Henne W. A., Doorneweerd D. D., Reifenberger R. G. & Low P. S. Detection of Bacillus subtilis spores using peptide-functionalized cantilever arrays. J Am Chem Soc 128, 3716–3721 (2006). 16536545 10.1021/ja0570887

[b32] Bai L. *et al.* Peptide-based isolation of circulating tumor cells by magnetic nanoparticles. J Mater Chem B Mater Biol Med 2, 4080–4088 (2014). 10.1039/c4tb00456f 32261739

[b33] Etayash H., Jiang K., Thundat T. & Kaur K. Impedimetric Detection of Pathogenic Gram-Positive Bacteria Using an Antimicrobial Peptide from Class IIa Bacteriocins. Anal Chem 86, 1693–1700 (2014). 24400685 10.1021/ac4034938

[b34] Mannoor M. S. *et al.* Graphene-based wireless bacteria detection on tooth enamel. Nat Commun 3, 763 (2012). 22453836 10.1038/ncomms1767

[b35] DeMello A. J. Control and detection of chemical reactions in microfluidic systems. Nature 442, 394–402 (2006). 16871207 10.1038/nature05062

[b36] Kolonin M. G. *et al.* Ligand-directed surface profiling of human cancer cells with combinatorial peptide libraries. Cancer Res 66, 34–40 (2006). 16397212 10.1158/0008-5472.CAN-05-2748

[b37] Rangel R. *et al.* Combinatorial targeting and discovery of ligand-receptors in organelles of mammalian cells. Nat Commun 3, 788 (2012). 22510693 10.1038/ncomms1773 PMC3337985

[b38] Chou J. *et al.* Effects of sample delivery on analyte capture in porous bead sensors. Lab chip 12, 5249–5256 (2012). 23117481 10.1039/c2lc40752c PMC3541674

[b39] Cunci L. *et al.* Real-Time Detection of Telomerase Activity in Cancer Cells using a Label-Free Electrochemical Impedimetric Biosensing Microchip. RSC Adv 4, 52357–52365 (2014). 25598969 10.1039/C4RA09689D PMC4295792

[b40] Khoo B. L. *et al.* Clinical validation of an ultra high-throughput spiral microfluidics for the detection and enrichment of viable circulating tumor cells. PLoS One 9, e99409 (2014). 24999991 10.1371/journal.pone.0099409 PMC4085042

[b41] Allard W. J. *et al.* Tumor cells circulate in the peripheral blood of all major carcinomas but not in healthy subjects or patients with nonmalignant diseases. Clin Cancer Res 10, 6897–6904 (2004). 15501967 10.1158/1078-0432.CCR-04-0378

[b42] Jacob K., Sollier C. & Jabado N. Circulating tumor cells: detection, molecular profiling and future prospects. Expert Rev Proteomics 4, 741–756 (2007). 18067413 10.1586/14789450.4.6.741

[b43] Lianidou E. S. & Markou A. Circulating tumor cells in breast cancer: detection systems, molecular characterization, and future challenges. Clin Chem 57, 1242–1255 (2011). 21784769 10.1373/clinchem.2011.165068

[b44] Lee S. K. *et al.* Nanowire substrate-based laser scanning cytometry for quantitation of circulating tumor cells. Nano Lett 12, 2967–2704 (2012). 10.1021/nl2041707 PMC3381426 22646476

[b45] Venkatanarayanan A., Keyes T. E. & Forster R. J. Lebel-free impedance detection of cancer cells. Anal Chem 85, 2216–2222 (2013). 23331159 10.1021/ac302943q

[b46] Halo T. L. *et al.* NanoFlares for the detection, isolation, and culture of live tumor cells from human blood. Proc Natl Acad Sci USA 111, 17104–17109 (2014). 25404304 10.1073/pnas.1418637111 PMC4260589

[b47] Lin H. K. *et al.* Portable filter-based microdevice for detection and characterization of circulating tumor cells. Clin Cancer Res 16, 5011–5018 (2010). 20876796 10.1158/1078-0432.CCR-10-1105 PMC2955786

[b48] Lee A. *et al.* All-in-one centrifugal microfluidic device for size-selective circulating tumor cell isolation with high purity. Anal Chem 86, 11349–11356 (2014). 25317565 10.1021/ac5035049

[b49] Arlett J. L., Myers E. B. & Roukes M. L. Comparative advantages of mechanical biosensors. Nat Nano 6, 203–215 (2011). 10.1038/nnano.2011.44 PMC3839312 21441911

[b50] Kenny L. M. *et al.* Phase I trial of the positron-emitting Arg-Gly-Asp (RGD) peptide radioligand 18F-AH111585 in breast cancer patients. J Nucl Med 49, 879–886 (2008). 18483090 10.2967/jnumed.107.049452

[b51] Fani M., Maecke H. R. & Okarvi S. M. Radiolabeled peptides: valuable tools for the detection and treatment of cancer. Theranostics 2, 481–501 (2012). 22737187 10.7150/thno.4024 PMC3364555

[b52] Rufini V., Calcagni M. L. & Baum R. P. Imaging of neuroendocrine tumors. Semin Nucl Med 36, 228–247 (2006). 16762613 10.1053/j.semnuclmed.2006.03.007

[b53] Etayash H., Norman L., Thundat T. & Kaur K. Peptide-bacteria interactions using engineered surface-immobilized peptides from class IIa bacteriocins. Langmuir 29, 4048–4056 (2013). 23445325 10.1021/la3041743

[b54] Harris J. R. Blood separation and plasma fractionation. (Wiley-Liss, 1991).

[b55] Jacobsen K. H. Introduction to health research methods: a practical guide. (Jones & Bartlett Learning, 2012).

