# Supplementary material for: Real-time Detection of Breast Cancer Cells Using Peptide-functionalized Microcantilever Arrays
Source: Sci Rep. 2015 Oct 5;5:13967. doi: 10.1038/srep13967 (PMC4593050; doi:10.1038/srep13967)
Supplement: Supplementary Information [file srep13967-s1.pdf]

SUPPLEMENTARY INFORMATION

Real-time Detection of Breast Cancer Cells Using Peptide-functionalized Microcantilever Arrays

Hashem Etayash[1,2], Keren Jiang[2], Sarfuddin Azmi[1], Thomas Thundat[2], and Kamaljit Kaur[1,3,*]

Table of Contents

Table S1. Short ligand peptide probes used in the study. S2

Figure S1. Schematic diagram of an in-house built microcantilever detection system. . S3

Figure S2. Capture yield as a function of flow rate (mL/h) from buffer solution spiked with MCF7 cells S4

Figure S3. Concentration dependent pattern of the peptide-based cantilever sensor S5

Figure S4. Differential deflection of microcantilever arrays with MCF7 spiked into human blood samples. S6

Table S1. Short ligand peptide probes used in the study.

Peptide	Targeting receptor	$[M+H]^{+1}$ Obs.	Calc.	Rt (min)	Yield %
**18-4** (WxEAAYQrFLC)	Unknown	1398.2	1397.5	26	61%
**Ref-1** (XEPAYQRFTC)	none	1225.2	1225.2	25	62%
cRGDfC	Integrin αvβ3	579.5	578.7	22	66%
**Ref-2** (cRADfC)	none	592.6	592.7	25	60%

RP-HPLC retention time (Rt) of synthetic peptides was measured using Vydac C18 analytical column with a gradient of (mobile phase 1 for peptides 1 and 2) 15 – 50% ACN/water (0.1% TFA) in 50 min with a flow rate of 1 mL min^{-1} or (mobile phase 2 for peptides 3 and 4) 15 – 55% ACN/water (0.1% TFA) in 50 min with a flow rate of 1 mL/min. Lower case letters denote D-amino acids. X is Nle (norleucine), and Ref indicates reference peptides for the targeting peptides.

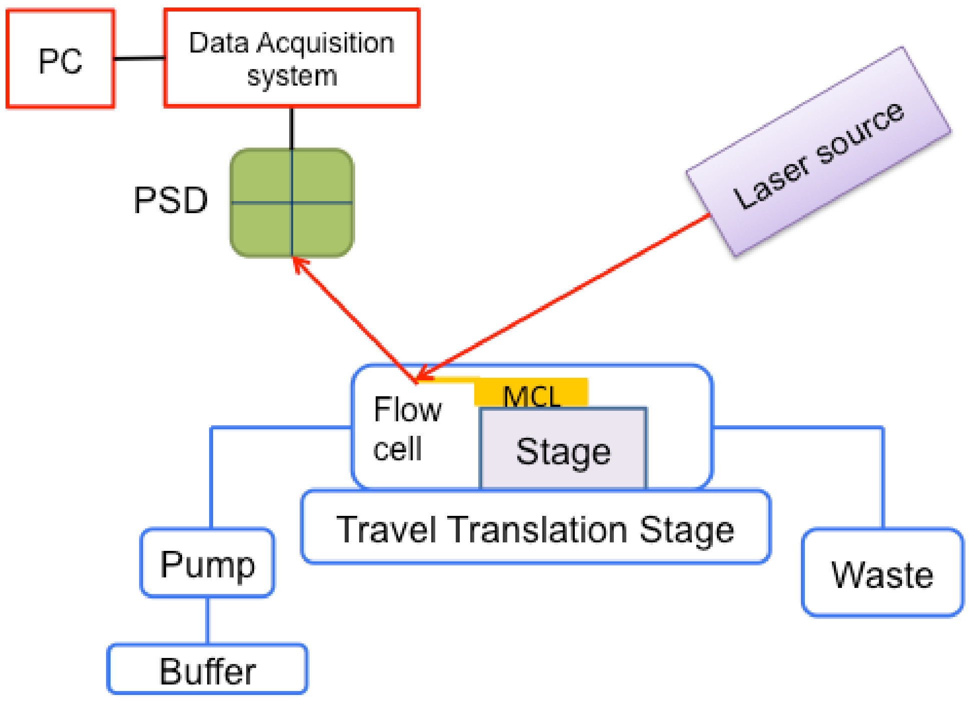

Figure S1. Schematic diagram of an in-house built microcantilever detection system. The parts for the sensor were purchased from Thorlabs. Inc (Newton, New Jersey, USA), while the levers were obtained from Concentris GmbH – Switzerland. The system consists mainly of the flow cell, where the cantilever is mounted to two ports, inlet and outlet, to receive and eject the delivered samples. Red diode laser beam reflect off the free end of the cantilever where the peptide is immobilized which then is focused on a position sensitive detector (PSD) to detect changes.

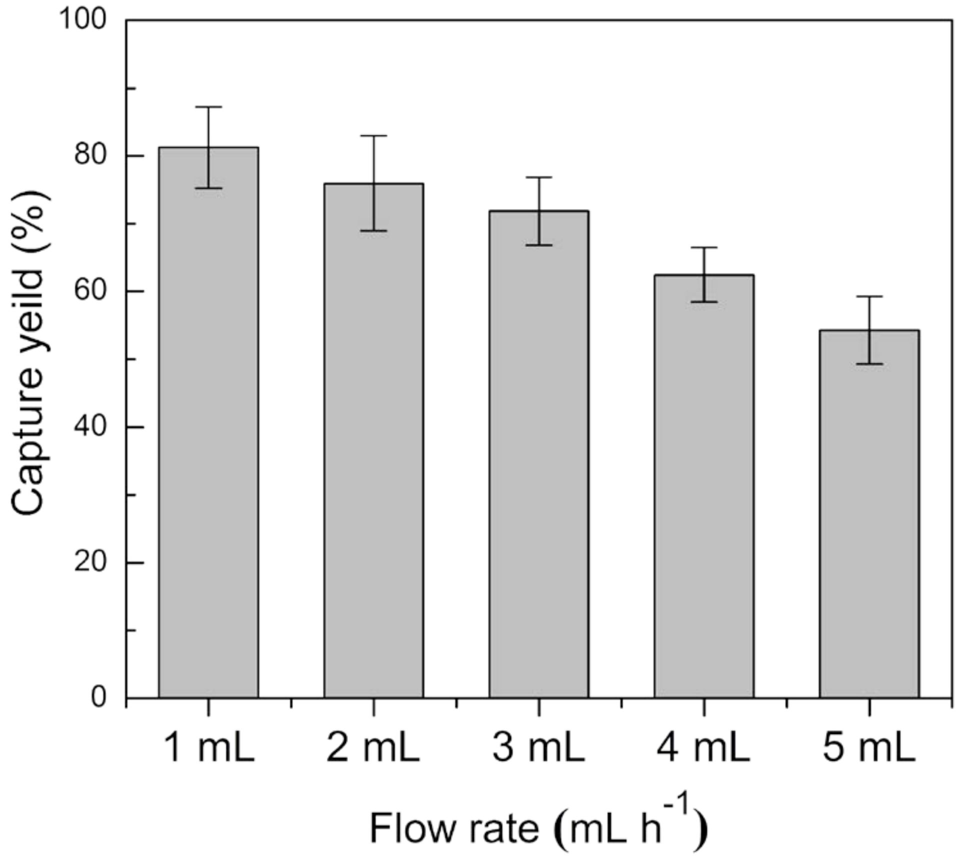

Figure S2. Capture yield as a function of flow rate (mL h-1) from buffer solution spiked with MCF7 cells (100 cells mL^{-1}). The responses were taken for a cantilever array sensor functionalized with peptide **18-4**. Each percentage is an average calculation of three replicates and error bars indicate corresponding standard deviations.

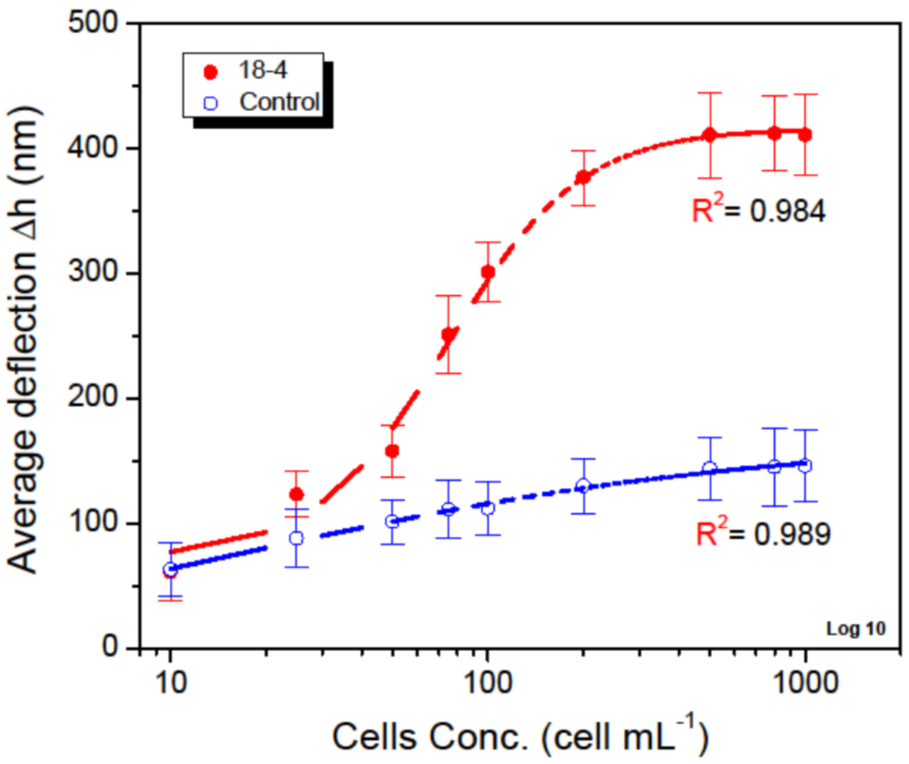

Figure S3. Concentration dependent pattern of the peptide-based cantilever sensor. The differential deflection of peptide **18-4** functionalized cantilever array (red) and control peptide cantilever (blue) was read after injection of serial concentration of MCF7 cancer cells spiked in phosphate buffered saline as indicated. The dashed lines show the sigmoid fit of the nanomechanical response as a function of MCF7 concentrations. Cantilever differential deflection represents an average calculation of eight replicates and error bars indicate standard deviations.

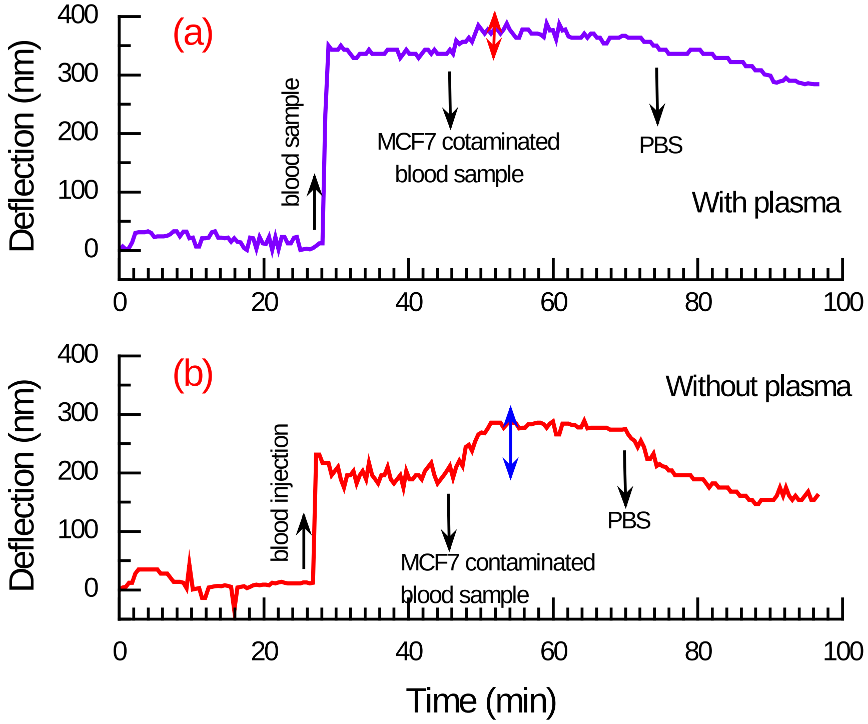

Figure S4. Differential deflection of microcantilever arrays with MCF7 spiked into human blood samples. (a) Top figure shows results from system equilibrated with PBS, followed by injection of whole blood and thereafter injection of MCF7 spiked blood (100 cancer cells/mL) and then again PBS, whereas (c) lower figure shows data from system equilibrated with PBS followed by injection of blood without plasma, and thereafter MCF7 spiked blood (100 cancer cells/mL) and again PBS.
